# KSHV *K15* genetic heterogeneity is associated with Kaposi sarcoma aggressiveness through modulation of the host inflammatory microenvironment

**DOI:** 10.1371/journal.ppat.1014615

**Published:** 2026-10-07

**Authors:** Yu Zhang, Peng Wang, Jingzhan Zhang, Yunying Wang, Tingting Li, Yuan Ding, Xiaojing Kang

**Affiliations:** 1 Department of Dermatology and Venereology, People’s Hospital of Xinjiang Uygur Autonomous Region, Urumqi, China; 2 Xinjiang Clinical Medical Research Center for Dermatology and Venereology, Urumqi, China; 3 Xinjiang Key Laboratory of Dermatology Research, Urumqi, China; Wistar Institute, UNITED STATES OF AMERICA

## Abstract

Kaposi sarcoma (KS) exhibits profound clinical heterogeneity, yet current risk assessment remains constrained by anatomical staging systems that lack universality across diverse KS variants. This study characterized the functional impact of Kaposi’s sarcoma-associated herpesvirus (KSHV) *K15* genotypes (M or P) on clinical aggressiveness in a retrospective cohort of 156 patients from Xinjiang, China. To enable unified analysis, we operationalized disease extent into a harmonized binary framework—categorized as localized or disseminated—by integrating anatomical criteria from the ACTG and Krigel classifications. Using causal mediation analysis to quantify the “Genotype–Inflammation–Edema” association, we identified the *K15* M-genotype (72.8%) was independently associated with a hyper-inflammatory phenotype. This revealed a Viral–Clinical Paradox: despite significantly lower HIV coinfection rates compared to P-carriers (10.3% vs. 27.5%; *P* = 0.009), M-carriers exhibited an elevated Systemic Immune-Inflammation Index (SII) and severe clinical edema. Mediation analysis confirmed that the SII significantly mediated 29.0% (*P* = 0.009) of the genotype-associated effect on edema. To translate these biological insights into clinical utility, a six-factor prognostic nomogram was developed using LASSO regression, maintaining an events-per-variable ratio of 11.2. The model demonstrated robust discrimination (Concordance Index, 0.761) and significantly outperformed traditional anatomical observation (AUC, 0.830 vs. 0.563; *P* = 0.003). Risk stratification successfully identified distinct subgroups, separating patients with rapid progression (median progression-free survival: 11.0 months) from those with favorable outcomes (40.0 months; *P* < 0.0001). Our results suggest that the *K15* M-genotype is associated with increased KS severity *via* intrinsic inflammatory activation, independent of host immunodeficiency. Integrating viral genotyping into clinical assessment may shift KS management from anatomical observation toward biology-driven precision oncology, identifying high-risk patients who may benefit from early systemic or anti-inflammatory interventions.

## Author summary

Kaposi sarcoma is a cancer caused by a viral infection. It affects patients in vastly different ways: some develop mild skin spots, while others suffer from rapidly growing tumors and swelling. Traditionally, doctors predict how a patient will fare by simply observing how far the tumors have physically spread. However, this visual method is often completely inaccurate. In our study, we asked whether small genetic differences in the virus itself could explain why some patients get much sicker than others. By analyzing 156 patients, we discovered that individuals infected with a specific genetic variant of the virus experienced a much more aggressive condition. We found that this specific viral variant is linked to severe, body-wide inflammation, which correlates with worse clinical outcomes, even in patients without underlying immune deficiencies. Using these biological insights, we developed a new risk-prediction tool that incorporates the genetics of the virus. We demonstrated that our biology-driven approach significantly outperforms traditional physical assessments. Our work suggests that routinely testing the virus’s genetics can help doctors precisely identify high-risk patients earlier, paving the way for targeted anti-inflammatory treatments to better manage this complex disease.

## Introduction

Kaposi sarcoma (KS) is a complex angioproliferative malignancy caused by the Kaposi’s sarcoma-associated herpesvirus (KSHV) [1]. Its clinical spectrum is remarkably heterogeneous, varying from indolent, slowly progressing cutaneous patches commonly seen in classic KS to aggressive, rapidly fatal visceral dissemination often associated with HIV coinfection [2]. In the modern era of widespread use of antiretroviral therapy, the classical paradigm linking KS progression to HIV-induced immunodeficiency has been fundamentally challenged. A significant subset of patients with an effectively reconstituted immune system experience rapid disease progression. This clinical observation highlights a potential “Viral-Clinical Paradox”, where aggressiveness persists despite immune reconstitution—suggesting that disease progression is associated not solely with host immunodeficiency but also with host genetic environmental factors and viral determinants, including *K15* heterogeneity and viral load [3].

Current paradigms in oncology have largely shifted toward precision medicine, where molecular subtyping dictates management, as has been successfully implemented in breast cancer and melanoma [4–6]. However, risk stratification for KS remains constrained by the reliance on anatomical staging systems such as the AIDS Clinical Trials Group (ACTG) and Krigel classifications [7–9]. Although valid for assessing the physical tumor burden, these frameworks are fundamentally limited because they capture the anatomical manifestations of the disease rather than its underlying biological factors. Consequently, they often fail to reliably predict individual outcomes, leaving clinicians without robust tools to distinguish between patients who require immediate systemic chemotherapy and those who are suitable for accumulation-sparing observation. There is a critical and unmet need for accessible biomarkers that reflect underlying pathogenic factors to guide precision management.

Tumor-promoting inflammation is increasingly recognized as a hallmark of cancer, particularly in virus-driven malignancies [10]. The genetic heterogeneity of KSHV is a potent but understudied determinant of clinical variability; however, its direct contribution to disease severity remains unproven [11,12]. The *K15* gene, located at the right end of the viral genome, encodes a multi-transmembrane signaling protein that exists as two highly divergent alleles: the prototype (P) form and minority (M) form [11–13]. These alleles share less than 33% amino acid homology in their cytoplasmic signaling domains, suggesting that they may use distinct molecular interactors relevant to pathogenesis. While the P-form has been extensively characterized for its ability to recruit Src-family kinases and TRAFs to activate pro-inflammatory and angiogenic pathways (NF-κB, MAPK) [14–16], the functional consequences of the M-form remain poorly understood in clinical settings. Despite this structural divergence, both variants are thought to converge in inflammatory signaling cascades. However, whether the M genotype is associated with a distinct clinical phenotype, specifically “inflammation-associated” aggressiveness, remains a critical knowledge gap, as most prior studies have been limited to descriptive molecular epidemiology without clinical correlation.

The Xinjiang region of China, a historical hub on the Silk Road, offers a unique window into this viral diversity owing to the high prevalence of KS and the co-circulation of distinct viral genotypes rarely seen in Western cohorts. Although the geographical prevalence of the *K15* genotype has been previously reported, data from Asia are limited and fragmented [11,12,17–22]. We hypothesized that the *K15* M-genotype may not merely be a neutral geographic marker but an associated factor in a specific “inflammatory phenotype” that observationally explains this viral-clinical paradox. This study aimed to (1) characterize the distribution of *K15* genotypes in the largest single-center KS cohort in Asia, (2) elucidate the potential biological pathways linking viral genotypes to clinical edema using causal mediation analysis, and (3) develop and validate a biologically informed prognostic nomogram to shift risk stratification from an anatomical stage to a biology-driven model.

## Results

### Baseline characteristics and distinct clinical phenotypes of KSHV *K15* genotypes

A total of 156 patients with KS were enrolled. After quality control and genotyping, 147 patients were included in the final analysis (*K15M*, n = 107 [72.8%]; *K15P*, n = 40 [27.2%]; Fig 1A). Although baseline demographics were comparable across groups (Table 1), distinct clinical profiles were observed (S1 Table). Notably, a divergence between HIV status and inflammatory burden was identified; despite a significantly lower prevalence of HIV coinfection in *K15*M carriers than in *K15P* carriers (10.3% vs. 27.5%, *P* = 0.009), the *K15M* group exhibited significantly elevated systemic inflammatory indices, including SII, neutrophil-to-lymphocyte ratio (NLR), and neutrophil counts (all *P* < 0.05; Figs 1B and 1C). Clinically, this profile was associated with a higher incidence of lower limb edema in *K15M* carriers (37.4% vs. 20.0%, *P* = 0.045). Conversely, mucosal involvement was significantly less frequent in the *K15M* group (5.6% vs. 17.5%, *P* = 0.044; Fig 1D).Furthermore, the proportional distribution of primary therapeutic modalities showed no significant difference between the *K15P* and *K15M* groups (*P* = 0.104, S2 Table), confirming that the subsequent survival analysis was not systematically biased by unbalanced therapeutic interventions.

**Table 1:** Baseline characteristics of patients with Kaposi sarcoma stratified by KSHV *K15* genotypes.

Characteristics	Total (N = 156)	*K15P* (N = 40)	*K15M* (N = 107)	SMD	*P*
**Demographics**					
Age, years, median [IQR]	64.0 [54.9–70.0]	63.5 [54.5–69.8]	65.0 [54.0–71.0]	0.066	0.747
Sex, male, n (%)	137 (87.8)	35 (87.5)	94 (87.9)	0.011	1.000
**Clinical Types of KS**				**0.426**	**0.016**
Classic KS, n (%)	123 (78.8)	25 (62.5)	90 (84.1)		
HIV-associated KS, n (%)	23 (14.7)	11 (27.5)	11 (10.3)		
Iatrogenic KS, n (%)	10 (6.4)	4 (10.0)	6 (5.6)		
**Clinical Features**					
Tumor stage				0.302	0.109
Localized, n (%)	76 (48.7)	15 (37.5)	56 (52.3)		
Disseminated, n (%)	80 (51.3)	25 (62.5)	51 (47.7)		
Only cutaneous, n (%)	138 (88.5)	33 (82.5)	97 (90.6)	0.239	0.244
Cutaneous and mucosal, n (%)	14 (9.0)	7 (17.5)	6 (5.6)	**0.375**	**0.044**
Cutaneous and visceral, n (%)	6 (3.8)	0	5 (4.7)	0.375	0.324
Lower limb edema, n (%)	52 (33.3)	8 (20.0)	40 (37.4)	**0.389**	**0.045**
Hypertension, n (%)	52 (33.3)	8 (20.0)	39 (36.4)	0.369	0.057
**Laboratory Parameters**					
White blood cell count (10^9^/L), median [IQR]	6.25 [5.00–7.84]	6.17 [4.45–7.90]	6.44 [5.02–7.84]	0.191	0.649
Platelet count (10^9^/L)	223.0 [178.0–259.0]	217.0 [167.5–244.0]	225.0 [185.0–265.0]	0.413	0.094
Neutrophil count (10^9^/L)	3.89 [3.08–4.86]	3.47 [2.73–4.08]	4.14 [3.20–5.13]	**0.522**	**0.003**
Lymphocyte count (10^9^/L)	1.58 [1.28–1.98]	1.89 [1.30–2.30]	1.54 [1.27–1.85]	**0.507**	**0.024**
SII, median [IQR]	566.8 [358.6–734.0]	392.8 [223.7–572.6]	622.6 [433.5–859.3]	**0.642**	**<0.001**
NLR, median [IQR]	2.52 [1.84–3.26]	1.94 [1.46–2.87]	2.61 [2.13–3.34]	**0.579**	**<0.001**
PLR, median [IQR]	140.2 [106.4–183.0]	111.5 [87.3–158.3]	146.5 [114.04–199.3]	**0.640**	**<0.001**
CD4 T-cell count (cells/μL), median [IQR]	607.5 [498.0–653.0]	604.5 [392.0–624.6]	607.7 [543.9–655.0]	0.021	0.389
Albumin, median [IQR]	36.7 [34.2–39.8]	36.9 [34.0–39.85]	36.6 [34.0–39.1]	0.025	0.991

**Fig 1:**
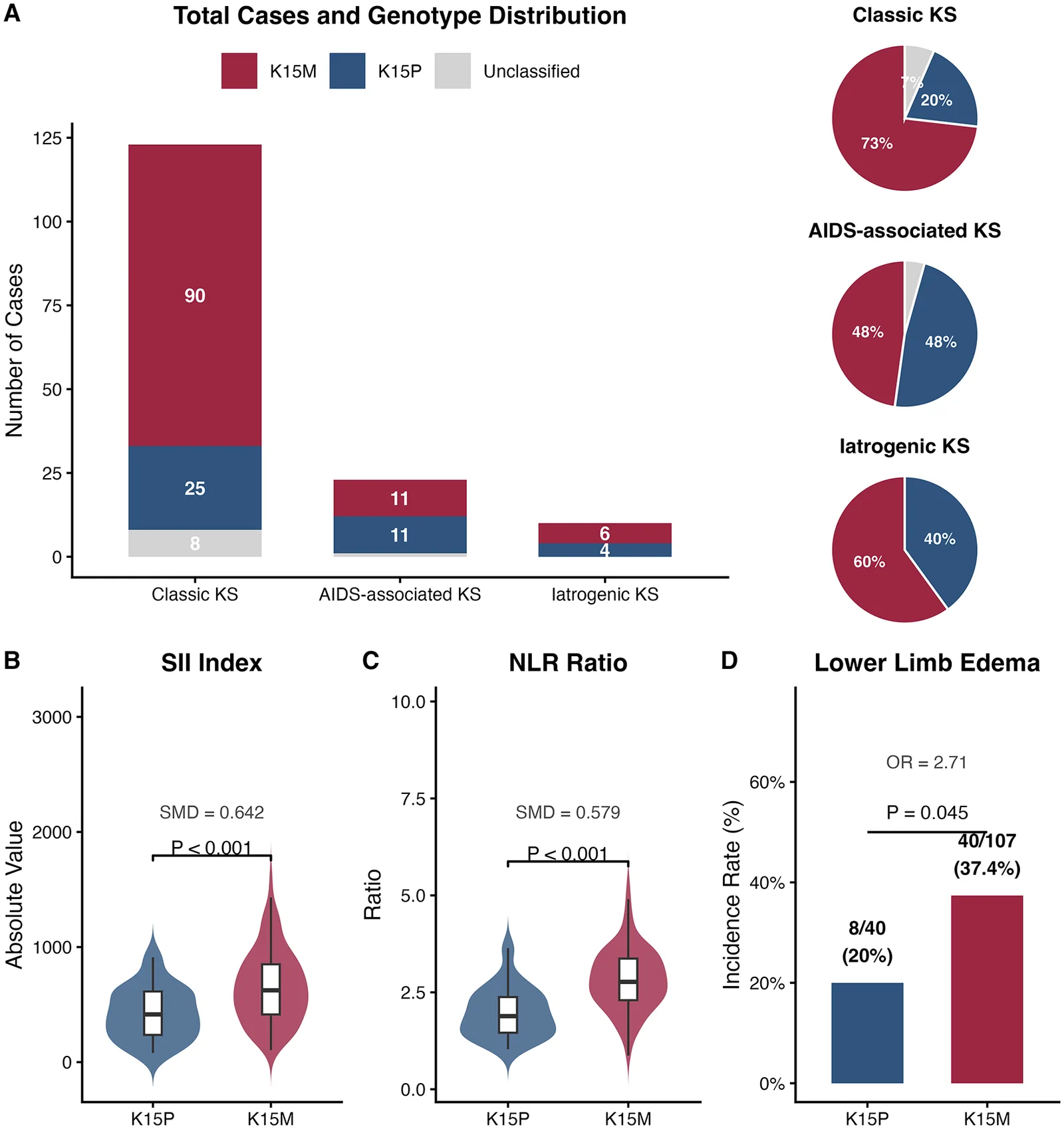
Prevalence of KSHV *K15* genotypes and associated inflammatory phenotypes. **(A)** Genotype distribution across clinical subtypes (N = 156). The stacked bar chart (left) and pie charts (right) illustrate the absolute and proportional prevalence of *K15M* (red), *K15P* (blue), and Unclassified (gray) strains, highlighting a *K15P* enrichment in AIDS-associated KS. **(B–C)** Inflammatory profiles compared between *K15M* (n = 107) and *K15P* (n = 40) carriers. Violin plots display the distribution of **(B)** SII and **(C)** NLR, with internal boxplots indicating median and IQR. *K15M* carriers exhibit a significantly higher inflammatory burden (*P* < 0.001). **(D)** Incidence of lower limb edema. Univariable OR = 2.71, *P* = 0.045 (unadjusted); multivariable-adjusted OR = 2.62, *P* = 0.034 (see main text and S1 Fig). Abbreviations: IQR, interquartile range; KS, Kaposi sarcoma; NLR, neutrophil-to-lymphocyte ratio; SII, Systemic Immune-Inflammation Index; SMD, standardized mean difference.

Data are presented as mean ± standard deviation (SD) for normally distributed variables, median [interquartile range, IQR] for skewed variables, or number (percentage) for categorical variables. The standardized mean difference (SMD) reflects the magnitude of imbalance between groups (SMD > 0.1 indicates a meaningful difference). *P*-values were calculated using Student’s t-test, Mann–Whitney U-test, or Chi-square test (χ²) as appropriate. Bold text indicates statistical significance (*P* < 0.05). The complete table can be found in Supplementary Material (S1 Table). Note: SII was calculated as (platelet count × neutrophil count)/ lymphocyte count. Tumor stage was defined as localized (skin confinement ± pure lymphadenopathy) vs. disseminated (visceral involvement, extensive edema, or ulceration) based on modified Krigel/ACTG criteria.Abbreviations: *K15P*, KSHV *K15* P-type; *K15M*, KSHV *K15* M-type; KS, Kaposi sarcoma; SII, systemic immune-inflammation Index; NLR, neutrophil-to-lymphocyte ratio; PLR, platelet-to-lymphocyte ratio.

### Multivariable analysis of genotype and phenotype

Multivariate logistic regression identified *K15M* carriage as an independent predictor of specific clinical phenotypes. Importantly, this association remained robust after adjusting for tumor stage and demographic factors (S1 Fig). Specifically, *K15M* carriers had 2.6-fold increased odds of lower limb edema (Model B: aOR = 2.62, 95% CI: 1.07–6.37, *P* = 0.034). Furthermore, this genotype strongly predicted a high inflammatory burden, including elevated SII (aOR = 5.43, *P* = 0.001), NLR, and PLR. Notably, these associations persisted regardless of the CD4 + T-cell count adjustment, suggesting that the viral genotype exerts a pathogenic effect independent of the patient’s baseline immunodeficiency.

### SII mediates *K15M*-associated edema via a genotype-dependent dose-response pathway

The results of causal mediation analysis implicated SII as a significant partial mediator linking *K15M* to edema (Fig 2A and S3A–C Tables). Although the direct effect of the genotype remained significant, the indirect pathway via SII was statistically robust (ACME *P* = 0.009), accounting for 29.0% of the total effect. To rigorously validate the directionality of this association, we performed a reverse mediation analysis (hypothesizing Edema → SII). Unlike the forward model, this reverse pathway failed to reach statistical significance (*P* = 0.075), supporting the hypothesis that inflammation may be associated with an upstream position in this association rather than a downstream consequence. Furthermore, the dose–response analysis revealed a distinctive genotype-dependent biologic gradient (Figs 2B and 2C). In *K15M* carriers, the risk of edema increased linearly with elevating SII (OR=1.58 per 500-unit increase, *P* = 0.025); no such relationship was observed in *K15P* carriers (*P* = 0.271), reinforcing that the inflammation–edema link is intrinsically tied to the viral genotype.

**Fig 2:**
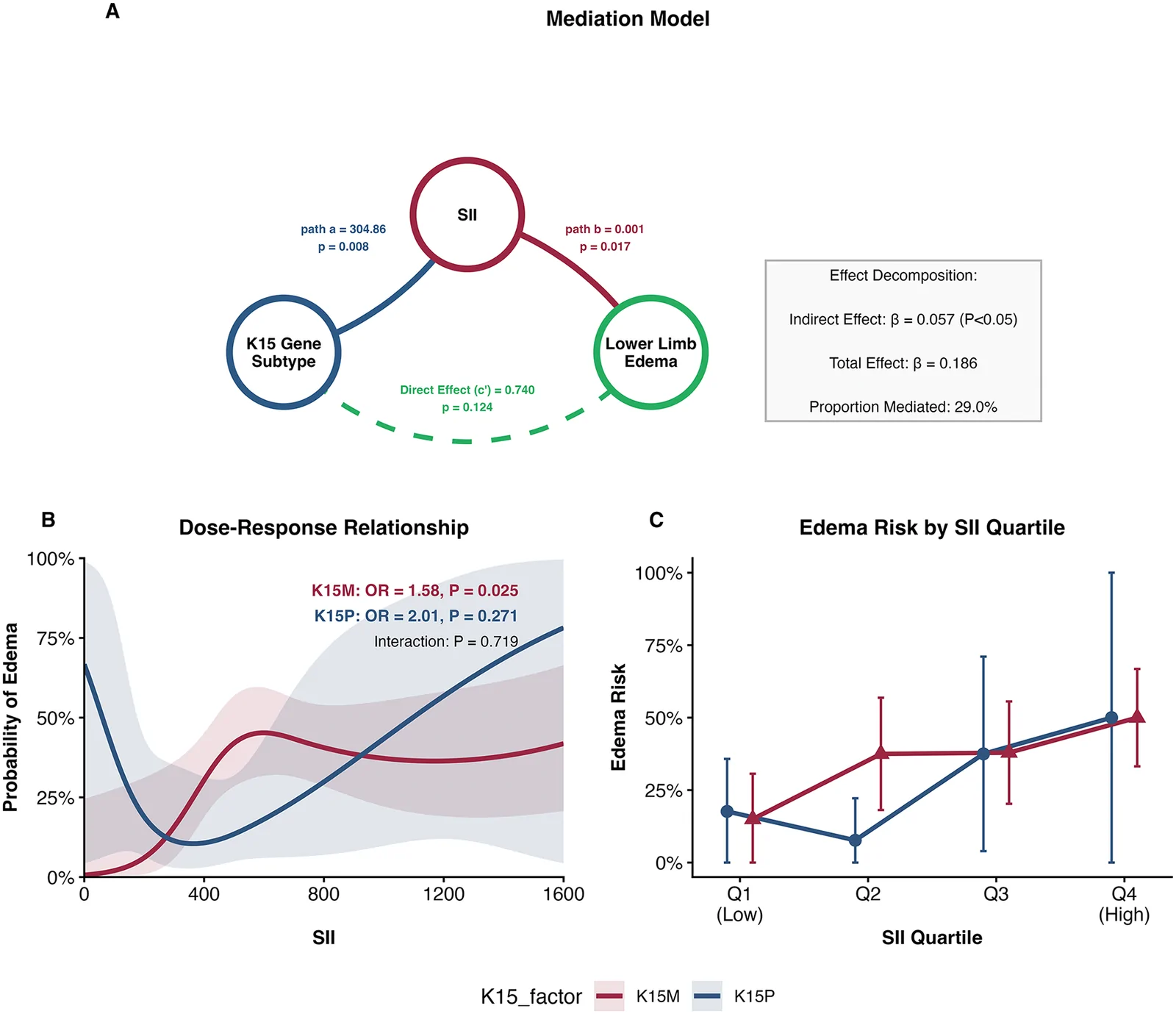
Comprehensive analysis of dose–response relationship between SII and lower limb edema risk. **(A)** Path diagram of mediation analysis. Visualization of the “Genotype–Inflammation (SII)–Edema” axis. The diagram displays coefficients and *P*-values for paths a, b, and **c.** The Effect Decomposition table highlights that the prognostic impact of *K15M* on edema is significantly mediated by systemic inflammation (ACME β = 0.057, *P* < 0.05; proportion mediated: 29.0%). Estimates were obtained using quasi-Bayesian Monte Carlo simulations (5,000 iterations). **(B)** Genotype-specific dose–response curves. A significant non-linear association is observed in *K15M* carriers (red line, *P* = 0.025) but not in *K15P* carriers (blue line, *P* = 0.271). Shaded areas represent 95% confidence intervals. **(C)** Edema risk stratified by SII quartiles. The plot shows the observed risk trend across increasing SII levels for each genotype. Error bars represent 95% confidence intervals. Abbreviations: ACME, average causal mediation effect; ADE, average direct effect.

#### *K15M* genotype as an independent prognostic factor for PFS.

Kaplan–Meier analysis demonstrated that *K15M* carriers experienced significantly inferior progression-free survival (PFS) (Log-rank *P* = 0.017; Fig 3A). In the multivariate Cox model, the *K15M* genotype retained a substantial hazard ratio (aHR = 1.87; Fig 3B). Although the *P*-value was borderline (*P* = 0.052), sensitivity analyses under varying censorship scenarios confirmed the robustness of this risk estimate (Fig 3C; S4 Table). Subgroup stratification revealed a stage-dependent pattern; the deleterious effect of *K15M* was most pronounced in localized disease (hazard ratio (HR) = 4.32, *P* = 0.009; Fig 3D and S2 Fig). Additionally, statistical interaction tests confirmed that age (using the sensitivity-validated 55-year cut-off; S3 Fig) did not confound the impact of the viral genotype (*P* for interaction = 0.555).

**Fig 3:**
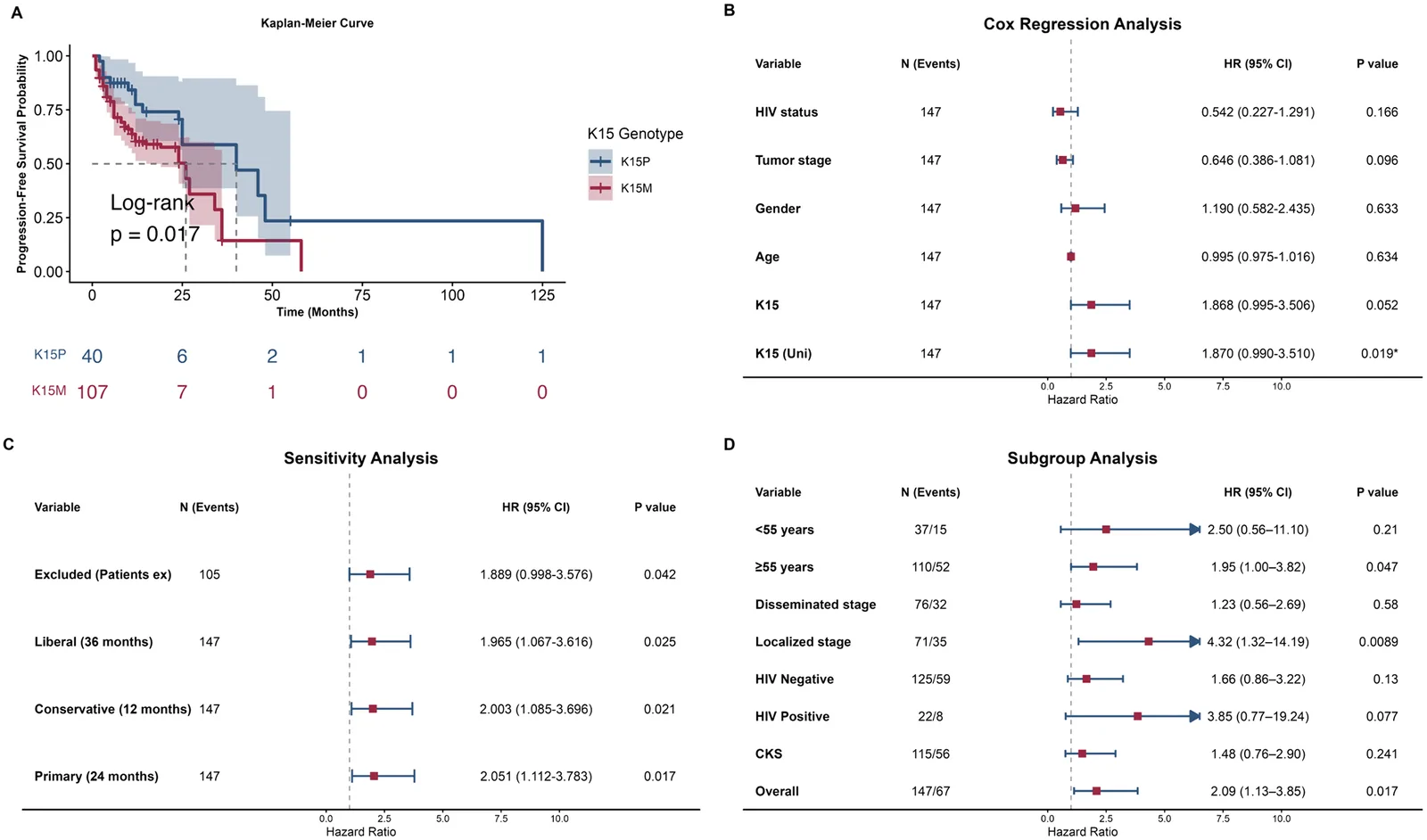
Impact of *K15M* genotype on progression-free survival (PFS). **(A)** Kaplan–Meier estimate. The curve demonstrates significantly reduced PFS in *K15M* carriers compared with that in *K15P* carriers (log-rank *P* = 0.017). **(B)** Forest plot of Cox regression analysis. Visualization of HR for *K15* genotype alongside clinical covariates (Age, Gender, Tumor Stage, and HIV status). The “*K15* (Uni)” row represents univariable results, while “*K15*” represents the multivariable adjustment. **(C)** Sensitivity analysis of progression-free survival (PFS). The forest plot illustrates the stability of the HR across four varying censorship scenarios (Primary, Conservative, Liberal, and Excluded). Consistent HRs (~2.0) confirm the robustness of the *K15M* genotype as a prognostic factor. Detailed statistics, including median PFS times for each scenario, are listed in S4 Table. **(D)** Subgroup analysis. Forest plot displaying the interaction between *K15* genotype and key clinical strata. The age threshold of 55 years was selected as the optimal stratification cut-off (S3 Fig). No significant interactions were observed (all interaction *P* > 0.05), suggesting that *K15M* is an independent risk factor across subgroups.

To assess whether the above associations were attributable to clinical subtype heterogeneity, we performed a sensitivity analysis restricted to Classic KS patients (n = 115; *K15P*, n = 25; *K15M*, n = 90). The inflammatory signature of *K15M* was consistently observed in this subgroup. *K15M* carriers exhibited significantly higher SII (*P* = 0.003) and NLR (*P* = 0.006) than *K15P* carriers. Associations with edema (aOR = 2.27, *P* = 0.304) and PFS (aHR = 1.44, *P* = 0.297; S4 Fig) remained directionally consistent but did not reach statistical significance, likely attributable to the limited *K15P* reference group (n = 25). The persistent inflammatory associations (*P* < 0.01) in this Classic KS subgroup do not support subtype-driven confounding as the primary explanation (S5 Table).

### Variable selection and hybrid model construction

Based on the optimized cut-off points derived from Maximally Selected Rank Statistics (MaxStat) (S6 Table), we implemented a systematic feature selection framework. In the Least Absolute Shrinkage and Selection Operator (LASSO)Cox regression, cross-validation identified core predictors with nonzero coefficients among the 12 candidates (S5C and S5D Figs). The final model (Table 2) utilized a hybrid strategy: the *K15* genotype and visceral status were forced into the model as biological anchors, whereas the CD4 count, PCT level, LMR, and NLR were selected based on LASSO strength. Notably, the non-linear prognostic association of baseline CD4 count was characterized using restricted cubic splines, identifying distinct high-risk phenotypes (S5E Fig). The SII was excluded to prevent collinearity redundancy (variance inflation factor < 1.10).

**Table 2:** Cox regression analysis results for model variable selection.

Variable	N	β	SE	Wald χ²	HR (95%CI)	*P*
*K15*	147	0.523	0.326	2.58	1.69(0.89,3.19)	0.108
Visceral involvement	147	0.873	0.541	2.61	2.40(0.83,6.91)	0.106
High CD4 count	21	1.412	0.345	16.73	4.10(2.09,8.07)	<0.001
Low PCT	32	0.822	0.288	8.13	2.27(1.29,4.00)	0.004
Low LMR	128	0.942	0.445	4.49	2.57(1.07,6.13)	0.034
Low NLR	69	-0.538	0.263	4.17	0.58(0.35,0.98)	0.041

Variables: Visceral involvement indicates patients with visceral organ involvement, distinct from localized cutaneous/mucosal disease (reference group). Cut-off determination: Optimal cut-offs for continuous variables were determined using MaxStat and dichotomized into binary variables (high vs. low) for the regression model. Specific cut-offs were: CD4^+^ T-cell count >792.69 cells/μL, PCT < 0.74 ng/mL, LMR < 1.27, and NLR > 2.48 (detailed in S6 Table). Model statistics: The final model was adjusted for multicollinearity and demonstrated robust fit (likelihood ratio test χ² = 42.9, *P* < 0.001). Abbreviations: HR, hazard ratio; CI, confidence interval; SE, standard error; LMR, lymphocyte-to-monocyte ratio; NLR, neutrophil-to-lymphocyte ratio; PCT, procalcitonin; PFS, progression-free survival.

### Validation and clinical utility of the nomogram

Based on these six predictors, a visual prognostic nomogram was constructed (Fig 4A). The nomogram demonstrated strong discriminative ability with a C-index of 0.761 (95% CI: 0.704–0.818) and excellent calibration (Fig 4B). Internal validation via bootstrapping (5,000 resamples) yielded a corrected C-index value of 0.743. Time-dependent receiver operating characteristic (ROC) curve analysis confirmed sustained accuracy over 1, 2, and 3 years (areas under the ROC curve [AUCs] > 0.80; S5F Fig). Subsequently, we benchmarked the nomogram against existing prognostic determinants. The proposed nomogram (AUC = 0.830) significantly outperformed both anatomical stage (AUC = 0.563, *P* = 0.003) and visceral involvement alone (AUC = 0.530, *P* < 0.001; Fig 4C). These gains were quantified statistically using reclassification metrics, which confirmed substantial improvements in risk-prediction accuracy (Integrated Discrimination Improvement

**Fig 4:**
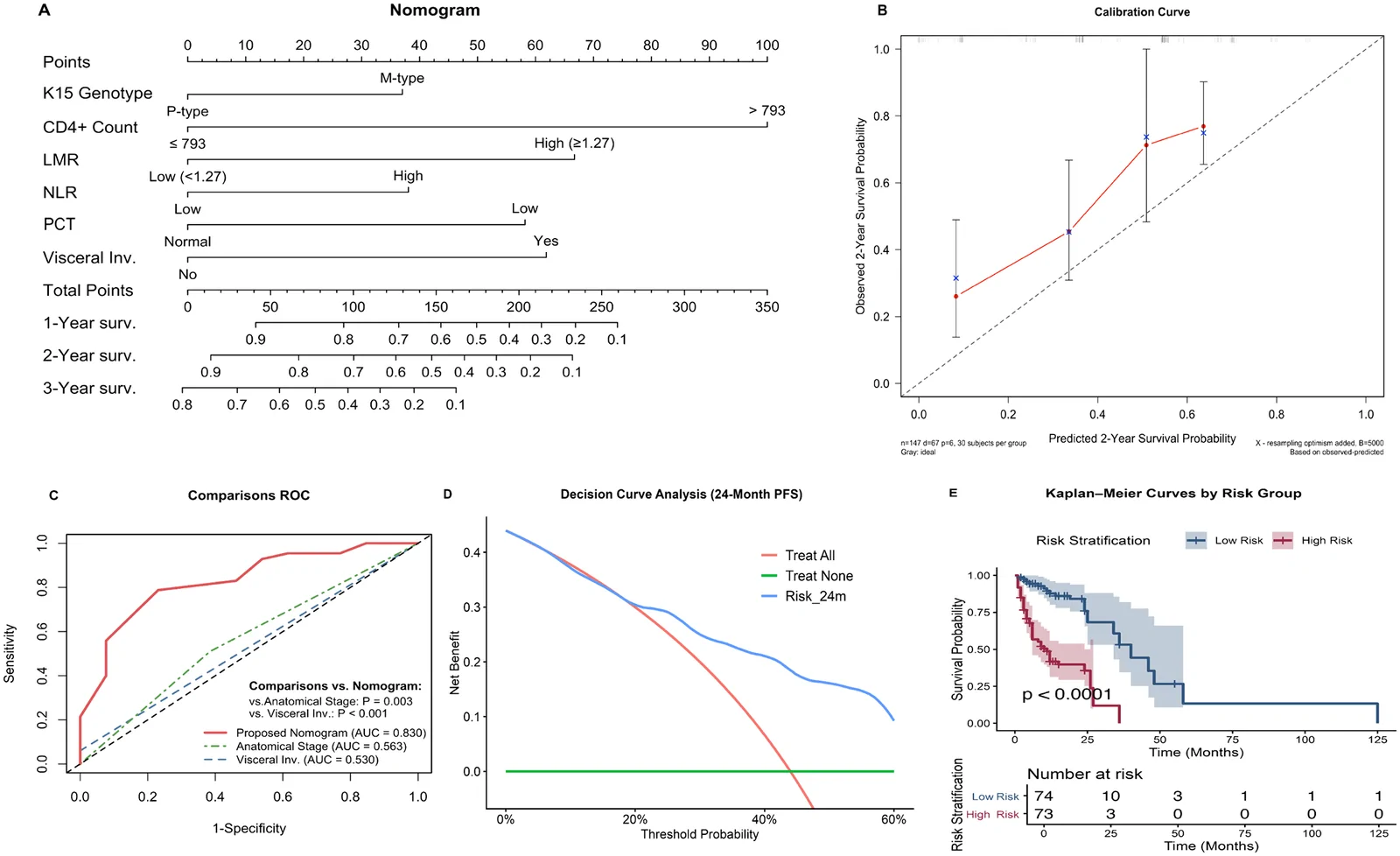
Construction and validation of the genotype-integrated prognostic nomogram. **(A)** Prognostic nomogram. The model integrates *K15* genotype, visceral involvement, CD4 count, LMR, NLR, and PCT. Points are assigned for each variable to estimate 1-, 2-, and 3-year PFS probabilities. **(B)** Calibration curve. The plot (based on 5,000 bootstrap resamples) demonstrates good agreement between predicted (x-axis) and observed (y-axis) survival probabilities at 2 years, aligning close to the ideal diagonal line. **(C)** Time-dependent ROC curve comparison. The proposed nomogram (red line, AUC = 0.830) demonstrates superior discriminative ability compared with anatomical stage (green line; localized vs. disseminated, AUC = 0.563) and visceral involvement (blue line, labeled as Visceral Inv.; AUC = 0.530). **(D)** Decision curve analysis (DCA). The curve confirms the net clinical benefit of using the nomogram for 24-month PFS prediction across a wide range of threshold probabilities. **(E)** Risk stratification. Kaplan–Meier curves stratifying patients into low-risk group vs. high-risk group based on the optimal nomogram score cut-off show significant separation (log-rank *P* < 0.0001).Abbreviations: AUC, area under the curve; LMR, lymphocyte-to-monocyte ratio; NLR, neutrophil-to-lymphocyte ratio; PCT,procalcitonin; PFS, progression-free survival; ROC, receiver operating characteristic.

(IDI) = 0.243, Net Reclassification Improvement Index (NRI) = 0.431; *P* < 0.001). The decision curve analysis (DCA) demonstrated a superior net clinical benefit across a wide range of threshold probabilities (Fig 4D). Finally, risk stratification based on the nomogram score successfully separated the patients into distinct prognostic groups (Median PFS: 11.0 vs. 40.0 months; *P* < 0.0001; Fig 4E). Crucially, subgroup analyses confirmed the model’s universal applicability: high-risk patients had significantly inferior PFS regardless of HIV status or tumor burden, with no significant interactions detected between the nomogram score and any stratification factor (all *P* > 0.05; S6 Fig).

## Discussion

In this retrospective cohort of 156 KS patients from Xinjiang, we observed that the *K15M* genotype is significantly associated with aggressive disease manifestations—including elevated systemic inflammation, edema, and reduced progression-free survival—despite lower HIV coinfection rates. These findings support a viral–clinical paradox, wherein disease severity appears dissociated from host immunodeficiency. While previous molecular epidemiological studies have mapped the global distribution of *K15* alleles [11,12,17–22], they have primarily focused on geographic prevalence. We describe a clinically relevant association: *K15M* carriers manifest a hyperinflammatory profile independent of traditional HIV-related immunosuppression. This observation challenges the dogma that aggressive KS is solely a consequence of host immune collapse and aligns with the hypothesis that the M-genotype may influence host signaling pathways to sustain systemic inflammation.

Historically, the Xinjiang region, a conduit of the Silk Road, represents a unique intersection of genetic diversity, where divergent viral strains co-circulate. The predominance of the M type (72.8%) in our cohort suggests that distinct evolutionary pressures may have been selected for this proinflammatory variant in Central Asian populations. This enrichment parallels that observed in classic KS cohorts from Japan and Taiwan and suggests host genetic factors may shape viral genotype distribution [19,22]. This geographic specificity underscores the limitations of generalizing prognostic models derived from Western cohorts to regions with distinct viral molecular epidemiology, highlighting the need for localized genomic surveillance.

A pivotal contribution of this study is the resolution of this paradox through causal mediation analysis to link viral genotype with clinical phenotype. Mechanistically, the biological plausibility of our statistical findings lies in the structural divergence of the *K15* protein. *K15* is a multi-pass transmembrane protein whose signaling capacity resides in its C-terminal cytoplasmic tail, which contains motifs (such as sources of SH2- and SH3-binding sites) that recruit Src-family kinases and TRAF adaptors to activate the NF-κB and MAPK pathways [23–25]. While the predominant P-type has been characterized to mobilize these pathways *via* specific tyrosine-based motifs, the M-type exhibits substantial sequence divergence (<33% homology) in this signaling domain [26,27]. Specifically, the M-type cytoplasmic tail exhibits reduced dependence on the canonical YEEV motif for NFAT activation [26], a feature that may permit constitutive signaling in diverse host microenvironments. We hypothesize that the M-type cytoplasmic tail may also harbor structurally distinct motifs that confer enhanced affinity for inflammatory adaptors or lack negative regulatory phosphorylation sites present in the P-type. Such structural variance would result in constitutive, unchecked signaling output. This aligns with our clinical observation that the M genotype is associated with sustaining high-grade systemic inflammation (SII). Our finding that SII mediates 29.0% of the genotype’s effect on edema is consistent with a pathogenic hierarchy: the viral signaling domain may be associated with the onset of the cascade, systemic inflammation may amplify it, and vascular instability (edema) may represent the clinical outcome. This model is biologically plausible given the structural divergence of the *K15* M-allele [26,27] but requires experimental validation. This is consistent with a potential feed-forward loop in which tumor-induced cytokines promote vascular permeability, facilitating stromal remodeling favorable for tumor progression.

This biological distinction has profound therapeutic implications, potentially paving the way for a “genotype-informed” therapy for KS. Currently, treatment guidelines are largely standardized across viral genotypes. However, our findings suggest that *K15M* carriers, who exhibit a distinct, likely NF-κB-driven inflammatory profile, may represent a subgroup with potential sensitivity to anti-inflammatory interventions. For these high-risk patients, traditional cytotoxic chemotherapy can be augmented with agents targeting the inflammatory axis, such as immunomodulators or specific cytokine inhibitors. While prospective trials are required, the *K15M* status could serve as a potential companion diagnostic tool to identify candidates for “anti-inflammatory” adjuvant strategies.

Translating these biological insights into clinical practice, we constructed a six-factor prognostic nomogram. In the model design, we balanced the mechanistic understanding with statistical stability; although SII was identified as a key mediator, it was excluded from the final nomogram to avoid multicollinearity, with NLR and LMR selected instead for their superior predictive independence. This validated nomogram represents a significant step toward precision medicine. Unlike the anatomical stage alone, which relies primarily on physical tumor distribution and may overlook the biological heterogeneity of early-stage disease, our model integrates viral genotyping with routine markers. With a concordance index of 0.761 and superior net clinical benefit demonstrated by DCA, this tool may help clinicians identify “high-risk localized” patients who may warrant early systemic intervention rather than the standard “watchful waiting” strategy.

This study has some limitations. First, this single-center retrospective design introduces potential selection bias, and the rarity of KS precluded an external validation cohort. The cohort showed marked subgroup imbalance, with *K15M* and classic KS being predominant—a pattern reported in other classic KS-endemic regions [19–22]. To address this, we confirmed model stability *via* internal bootstrapping (5,000 replicates) and subgroup analyses, and the event-per-variable ratio (11.2) exceeded the recommended threshold (>10) for multivariable modeling. Furthermore, to mitigate the potential confounding effect of clinical subtype heterogeneity, a dedicated sensitivity analysis confined to Classic KS patients (n = 115) confirmed that the core inflammatory associations of *K15M* (SII and NLR, both *P* < 0.01) were preserved independently of KS subtype, although clinical endpoints (edema and PFS) did not reach statistical significance due to the limited *K15P* reference group (n = 25) in this subgroup (S4 Fig, S5 Table).Second, a small proportion (5.8%) of viral genotypes remained unclassified. This may reflect either (i) recombination events between P and M alleles—a phenomenon documented in other herpesviruses—or (ii) the presence of *K15* variant(s) not covered by our PCR primers, such as the reported N-type (or non-P/M) alleles [21]. Future whole-genome sequencing will be needed to resolve this ambiguity. Third, this study lacks baseline epidemiological data on the prevalence of *K15* genotypes in the general Xinjiang population or in asymptomatic KSHV carriers, and therefore we cannot directly demonstrate relative enrichment of *K15M* among KS patients in this region. Population-based epidemiological surveys will be required in the future to formally validate this observation. Fourth, the assessment of edema in this study was performed using qualitative rather than quantitative operational definitions. As with any retrospective observational study, residual unmeasured confounding cannot be fully excluded by available covariates. The results of the causal mediation analysis should therefore be interpreted as an exploratory, biologically plausible association rather than a definitive causal inference. Finally, our evidence linking the *K15M* genotype to a hyper-inflammatory phenotype relies on clinical inflammatory indices, statistical associations, and causal mediation analysis, rather than direct wet-lab experimentation. While archival plasma was utilized for viral DNA genotyping—a process involving highly stable nucleic acids—these long-term retrospective samples lacked the strictly standardized pre-analytical handling necessary for sensitive protein-level cytokine arrays. As a result, running direct molecular assays to biologically prove the exact inflammatory markers induced by *K15M* in this historical cohort would be prone to significant artifacts. Thus, although our statistical findings are biologically plausible and conceptually aligned with existing *in vitro* studies of *K15* signaling variations [25,27], future investigations employing strictly controlled prospective cohorts and transcriptomic profiling (RNA sequencing) are essential to confidently map the precise downstream cytokine cascades driving this phenotype.

In summary, the KSHV *K15M* genotype was significantly associated with KS aggressiveness in this cohort, suggesting a proinflammatory pathway independent of host immunodeficiency. We validated a genotype-integrated nomogram that significantly outperforms anatomical staging. While these findings warrant prospective evaluation, they provide a strong rationale for incorporating *K15* genotyping into the clinical assessment of KS in regions with high M-genotype prevalence, particularly to identify patients who may benefit from early anti-inflammatory strategies.

## Materials and methods

### Ethics statement

The requirement for informed consent was waived because of the retrospective nature of the analysis, and patient data were anonymized prior to access. The study was approved by the Institutional Ethics Committee of the Xinjiang Uygur Autonomous Region People’s Hospital (ΚΥ2024101008). The individual in the clinical photograph (S8 Fig) has given written informed consent (as outlined in PLOS consent form) to publish this image under a Creative Commons license.

### Study population and design

This retrospective cohort study included 156 patients with histopathologically confirmed KS who were admitted to Xinjiang Uygur Autonomous Region People’s Hospital (January 2007–June 2025). The hospital is a tertiary center serving a diverse population in Northwest China; thus, the study cohort is representative of the population and viral epidemiology of the region. The inclusion criteria were as follows: (1) histopathologically confirmed KS; (2) availability of pretreatment peripheral blood of sufficient quality for genomic DNA extraction; and (3) ascertainable clinical outcomes with ≥6 months follow-up, except for patients who reached primary endpoints (progression or death) earlier. Exclusion criteria were: (1) incomplete clinical data preventing accurate staging or outcome assessment; and (2) age < 18 years. The study adhered to the Strengthening the Reporting of Observational Studies in Epidemiology (STROBE) guideline for observational reporting [28] and the Transparent Reporting of a Multivariable Prediction model for Individual Prognosis or Diagnosis (TRIPOD) guidelines for prognostic models [29] (S1 and S2 Files).

### KSHV genotyping

Genomic DNA was extracted from peripheral blood mononuclear cells (PBMCs) using a Polymeric Nucleic Acid Extraction Kit (MF061–04; Mei5 Biotechnology Co, Ltd, Beijing, China) following the manufacturer’s protocol. *K15* genotyping was performed using highly sensitive subtype-specific nested polymerase chain reaction (PCR) assays targeting distinct variable exons of KSHV *K15*. The complete list of primer sequences based on previous protocols [11,19,30] is shown in S7 Table. To ensure assay validity, the PCR runs included strict negative (water) and positive controls. Amplicons were visualized via 1.5% agarose gel electrophoresis, with genotypes assigned based on the band size: 285 bp for the P-type and 370 bp for the M-type (S7 Fig). Assay specificity was further validated by Sanger sequencing of representative products (GenBank reference NC_009333.1) [21]. Samples failing to amplify with either primer set after three independent attempts were classified as “Unclassified” and excluded from genotype-specific analyses.

### Outcomes, definitions

To address the absence of a universal staging system encompassing all KS variants, we operationalized disease extent into a harmonized binary framework to enable unified analysis. Patients were stratified independently of etiology based on phenotypic severity, integrating anatomical criteria from the Krigel and ACTG classifications [7,8]. Localized disease was defined as the presence of cutaneous lesions with or without non-bulky regional lymphadenopathy or minimal (non-nodular) oral involvement (equivalent to Krigel I/II and ACTG T0). Disseminated disease was characterized by visceral involvement, extensive oral disease, tumor-associated edema, and ulceration (equivalent to Krigel III/IV and ACTG T1). Given the retrospective nature of the historical EMR data, tumor-associated edema was operationalized as a binary clinical variable (present vs. absent). Tumor-associated edema was defined qualitatively as visually apparent, non-physiologic tissue swelling secondary to KS lesions relative to the patient’s normal baseline or contralateral limb, rather than by standardized quantitative metrics. Representative anonymized clinical photographs illustrating typical KS-associated edema in this cohort are provided in S8 Fig. Given the 18-year span and resultant therapeutic heterogeneity, specific treatment regimens were not included as covariates. Instead, to evaluate potential treatment-induced bias between virological groups, primary interventions were categorized into three main tiers (systemic or combined therapy, local therapy, and no active treatment) and independently compared between *K15* genotypes. This strategy prioritizes isolating intrinsic viral and host drivers for initial risk stratification, independent of subsequent interventions. The primary endpoint, PFS, was defined as the time from diagnosis to progression (RECIST 1.1), death, or last follow-up. A validated 24-month censoring interval was applied to account for irregular surveillance.

### Statistical analysis

Continuous and categorical variables were compared using Mann–Whitney U and Chi-square tests. Optimal survival cut-offs for inflammatory indices were determined using MaxStat to maximize stratification, as standard ROC curve analysis lacked discriminative power (S5A and S5B Figs). For age, sensitivity analysis supported a clinical threshold of 55 years (S3 Fig) over the MaxStat-derived 69 years. Non-linear relationships were evaluated using restricted cubic splines (S5C Fig). The “Genotype–Inflammation–Edema” association was investigated using causal mediation analysis. Model selection employed a hybrid framework: (1) LASSO Cox regression with 10-fold cross-validation (S5D and S5E Figs); and (2) forced inclusion of genotype and visceral status. The proportional hazards assumption for the Cox regression was verified using Schoenfeld residuals (global test *P* = 0.164, with all individual covariates *P* > 0.05). Collinearity was assessed via VIF. The final model maintained an EPV of 11.2. Performance and incremental utility were evaluated using the C-index (bootstrapped 5,000 times), calibration plots, time-dependent ROC (S5F Fig), DCA, NRI, and IDI. Consistency was verified across subgroups (S6 Fig). To evaluate the potential influence of KS clinical subtype on the observed genotype–phenotype associations, a pre-specified sensitivity analysis was conducted in the Classic KS subgroup (n = 115 with available *K15* genotyping data), examining inflammatory indices (Wilcoxon rank-sum test), lower limb edema (univariable and multivariable logistic regression), and PFS (multivariable Cox regression and Kaplan–Meier analysis), with the same covariate adjustment structure applied to the primary analyses. Statistical analyses were performed using R (v4.5.2), and two-sided *P* < 0.05 was considered significant.

## Supporting information

S1 Table Univariate Associations between 39 Clinical/Laboratory Parameters and *K15* Genotypes. This table presents the distribution of comorbidities, clinical features, and laboratory parameters stratified by *K15* genotype (P vs. M). Statistical significance was assessed using Chi-square or Mann–Whitney U tests as appropriate.
(DOCX)

S2 Table Distribution of Primary Treatment Modalities Across KSHV *K15* Genotypes. This table details the initial therapeutic interventions. Statistical analysis (Fisher’s exact test) was used to assess treatment balance between *K15P* and *K15M* groups, ensuring that observed prognostic differences are not primarily driven by treatment bias.
(DOCX)

S3 Table Causal Mediation Analysis, Directionality, and Dose-Response Assessment Linking *K15M* Genotype to Lower Limb Edema. Panel A compares mediation effects of SII, NLR, and PLR. Panel B shows sensitivity analyses adjusting for CD4 counts. Panel C validates directionality via reverse mediation. Panel D quantifies genotype-specific dose-response relationships between SII and edema.
(DOCX)

S4 Table Sensitivity Analysis of Progression-Free Survival Under Varying Censoring Scenarios. The table reports median PFS and hazard ratios under primary (24 months), conservative (12 months), liberal (36 months), and excluded-patient scenarios, confirming the robustness of the *K15M* prognostic effect.
(DOCX)

S5 Table Associations of KSHV *K15* Genotypes with Clinical, Inflammatory, and Prognostic Features in the Classic KS Subgroup. The table summarizes the comparison of inflammatory indices (SII and NLR) via Wilcoxon rank-sum tests, alongside univariable and multivariable logistic regression for lower limb edema, and multivariable Cox proportional hazards regression for PFS. Both multivariable models were adjusted for age, sex, and tumor stage. Analyses were strictly restricted to Classic KS patients with available *K15* genotyping data (N = 115).
(DOCX)

S6 Table Variable selection process and multicollinearity assessment for the prognostic model. Stepwise details include MaxStat optimal cutoffs, univariate hazard ratios, LASSO coefficients at λ_min_, and variance inflation factors (VIF) for candidate variables. Variables with nonzero LASSO coefficients were retained in the final model.
(DOCX)

S7 Table Primer sequences used for KSHV *K15* genotyping via nested PCR. The table lists primer names, sequences (5′ → 3’), amplicon sizes, and original references for both P-type and M-type specific PCR rounds.
(DOCX)

S1 Fig Robustness of the association between *K15* genotype and clinical/inflammatory phenotypes across adjustment models. Forest plot displaying adjusted odds ratios (aOR) for *K15M* vs. *K15P* carriers. Model A adjusts for age, sex, tumor stage, and HIV status; Model B additionally adjusts for CD4^+^ T-cell count. Error bars indicate 95% confidence intervals.
(TIF)

S2 Fig Effect of *K15* Genotype on PFS by Subgroups. Kaplan–Meier curves and hazard ratio forest plots illustrating the prognostic impact of *K15M* across different clinical subgroups, including age, sex, HIV status, and tumor stage.
(TIF)

S3 Fig Impact of Age on Stratification and Survival. (a) Age distribution histogram stratified by *K15* genotype. (b) Sensitivity analysis assessing the stability of *K15M* hazard ratio across varying age cutoffs (50–70 years). The optimal threshold (55 years) was selected based on HR stability and statistical significance.
(TIF)

S4 Fig Kaplan-Meier estimates of progression-free survival in the Classic Kaposi Sarcoma subgroup, stratified by KSHV *K15* genotypes. Survival curves display outcomes for *K15M* (red, n = 90) versus *K15P* (blue, n = 25) carriers among genotyped Classic KS patients (N = 115). Shaded regions represent 95% confidence intervals. A number-at-risk table is aligned below the x-axis. The log-rank test yielded a *P*-value of 0.24, with the non-significant outcome reflecting the attenuated statistical power attributable to the limited sample size of the reference *K15P* subgroup.
(TIF)

S5 Fig Statistical methodology for variable selection and model construction. (a–b) Comparison of ROC-derived vs. MaxStat-derived cutoffs using SII. (c) Restricted cubic spline showing nonlinear relationship between CD4 count and PFS. (d–e) LASSO coefficient paths and cross-validation for tuning parameter λ. (f) Time-dependent ROC curves for 1-, 2-, and 3-year PFS predictions.
(TIF)

S6 Fig Subgroup analysis of the prognostic nomogram for progression-free survival. Forest plot displaying hazard ratios (HR) and 95% confidence intervals for high-risk vs. low-risk groups across clinical stratifications. The nomogram’s predictive performance is consistent across all subgroups.
(TIF)

S7 Fig *K15* genotype-specific PCR electrophoresis results. Lane M: DNA marker (100–2000 bp); Lane 1: *K15P* positive control (285 bp); Lane 2: *K15M* positive control (370 bp). Representative gel image confirming genotype-specific amplification.
(TIF)

S8 Fig Representative clinical photographs of tumor-associated edema in Kaposi Sarcoma patients. (a-b) Visually apparent tissue swelling adjacent to lower-extremity KS lesions, which was clinically assessed relative to the patient’s normal baseline.
(TIF)

S1 Raw Image Raw image file for S7 Fig (full-size unedited gel image). (TIF)

S1 File STROBE Statement. Checklist of items that should be included in reports of cohort studies, completed for this study.This checklist is adapted from the STROBE Statement (https://www.strobe-statement.org/), licensed under CC BY 4.0 (https://creativecommons.org/licenses/by/4.0/).
(PDF)

S2 File TRIPOD Checklist. Checklist for prediction model development and validation, completed for this study.This checklist is adapted from the TRIPOD Statement (https://www.tripod-statement.org/), licensed under CC BY 4.0 (https://creativecommons.org/licenses/by/4.0/).
(PDF)
