# Supplementary material for: KSHV K15 genetic heterogeneity is associated with Kaposi sarcoma aggressiveness through modulation of the host inflammatory microenvironment
Source: PLoS Pathog. 2026 Oct 7;22(10):e1014615. doi: 10.1371/journal.ppat.1014615 (PMC13660985; doi:10.1371/journal.ppat.1014615)
Supplement: Supplementary file 1 [file ppat.1014615.s017.pdf]

S1 File: STROBE Statement—Checklist of items that should be included in reports of ***cohort studies***

	Item No	Recommendation	Page No.	Relevant text from manuscript
**Title and abstract**	1	(*a*) Indicate the study's design with a commonly used term in the title or the abstract	2	Abstract (contains "Nomogram" implying cohorexplicitly states "retrospective cohort study").
		(*b*) Provide in the abstract an informative and balanced summary of what was done and what was found	2	Abstract section (Background, Methods, Results, Conclusion).
**Introduction**				
Background/rationale	2	Explain the scientific background and rationale for the investigation being reported	4	Introduction (Paragraphs discussing KS, K15 genotype background, and the need for new prognostic tools).
Objectives	3	State specific objectives, including any prespecified hypotheses	6	Introduction (Last paragraph: "This study aimed to visualize the prognostic value... by developing a nomogram...").
**Methods**				
Study design	4	Present key elements of study design in the paper	23	Materials and Methods - Subsection "Study Population and Design" (States "retrospective cohort study").
Setting	5	Describe the setting, locations, and relevant dates, including periods of recruitment, exposure, follow-up, and data collection	23	Materials and Methods - "Study Population and Design" (Xinjiang People's Hospital, Jan 2007–June 2025).
Participants	6	(*a*) Give the eligibility criteria, and the sources and methods of selection of participants. Describe methods of follow-up	23-24	Materials and Methods- "Study Population and Design" (Inclusion criteria 1-3; Exclusion criteria 1-2).
		(*b*) For matched studies, give matching criteria and number of exposed and unexposed	N/A	Not applicable, this is not a matched study
Variables	7	Clearly define all outcomes, exposures, predictors, potential confounders, and effect modifiers. Give diagnostic criteria, if applicable	25	Materials and Methods- "Data Collection" & "Follow-up" (Defines PFS, K15 genotypes, SII, NLR, clinical stages).
Data sources/ measurement	8*	For each variable of interest, give sources of data and details of methods of assessment (measurement). Describe comparability of assessment methods if there is more than one group	*24*	Materials and Methods - "KSHV Genotyping" (PCR details) & "Data Collection" (Medical records review).
Bias	9	Describe any efforts to address potential sources of bias	23-24	Materials and Methods - "Study Population" (Selection criteria standardized to reduce bias; Retrospective nature acknowledged).

Study size	10	Explain how the study size was arrived at	23-24	Materials and Methods- "Study Population" (All eligible patients in the defined period: 156 cases).
Quantitative variables	11	Explain how quantitative variables were handled in the analyses. If applicable, describe which groupings were chosen and why	26	Materials and Methods - "Statistical Analysis" (Describes categorization of SII, NLR using X-tile or ROC cutoffs).
Statistical methods	12	(*a*) Describe all statistical methods, including those used to control for confounding	26	Materials and Methods- "Statistical Analysis" (Cox regression, LASSO, Log-rank test).
		(*b*) Describe any methods used to examine subgroups and interactions	26	Materials and Methods - "Statistical Analysis" (Mention of subgroup analysis).
		(*c*) Explain how missing data were addressed	24	Materials and Methods - Exclusion criteria (Excluded patients with incomplete data).
		(*d*) If applicable, explain how loss to follow-up was addressed	25	Materials and Methods - Censoring rules described in outcome definition.
		(*e*) Describe any sensitivity analyses	26	Materials and Methods - "Statistical Analysis" (Mention of sensitivity analysis).
**Results**				
Participants	13*	(a) Report numbers of individuals at each stage of study—eg numbers potentially eligible, examined for eligibility, confirmed eligible, included in the study, completing follow-up, and analysed	6	Results - "Patient Characteristics" (156 enrolled -> 9 unclassified -> 147 final cohort).
		(b) Give reasons for non-participation at each stage	6	Results - "Patient Characteristics" (9 patients excluded due to "Unclassified" K15 genotyping results).
		(c) Consider use of a flow diagram	N/A	Cited in Results (patient selection).
Descriptive data	14*	(a) Give characteristics of study participants (eg demographic, clinical, social) and information on exposures and potential confounders	6-7	Results - "Patient Characteristics" & Table 1 (Demographic and clinical baseline).
		(b) Indicate number of participants with missing data for each variable of interest	6	Results (Mentioning the specific number analyzed; missing data led to exclusion).
		(c) Summarise follow-up time (eg, average and total amount)	6	Results (Median follow-up time reported).
Outcome data	15*	Report numbers of outcome events or summary measures over time	7-8	Results - "K15 Genotypes... Survival" (Number of progression events, median PFS).
Main results	16	(*a*) Give unadjusted estimates and, if applicable, confounder-adjusted estimates and their precision (eg, 95% confidence interval). Make clear which confounders were adjusted for and why they were included	13	Results - "Predictors of PFS" & Figure 3 (Uni- and Multivariate Cox regression HRs).
		(*b*) Report category boundaries when continuous variables were categorized	8	Results (Thresholds for SII, NLR, etc., stated).

		(*c*) If relevant, consider translating estimates of relative risk into absolute risk for a meaningful time period	16	Results (Nomogram predicts 1-, 2-, 3-year survival probabilities).
Other analyses	17	Report other analyses done—eg analyses of subgroups and interactions, and sensitivity analyses	16	Results - "Validation and Clinical Utility" (Subgroup analysis, DCA, Sensitivity analysis).
**Discussion**				
Key results	18	Summarise key results with reference to study objectives	18	Discussion -First paragraph
Limitations	19	Discuss limitations of the study, taking into account sources of potential bias or imprecision. Discuss both direction and magnitude of any potential bias	21-22	Discussion - (Retrospective nature, single-center, sample size).
Interpretation	20	Give a cautious overall interpretation of results considering objectives, limitations, multiplicity of analyses, results from similar studies, and other relevant evidence	21	Discussion (Comparison with other studies, clinical implication of the nomogram).
Generalisability	21	Discuss the generalisability (external validity) of the study results	23	Discussion (Mentioning the representative nature of the Northwest China cohort).
**Other information**				
Funding	22	Give the source of funding and the role of the funders for the present study and, if applicable, for the original study on which the present article is based	29	Funding section.

*Give information separately for exposed and unexposed groups.

Note: An Explanation and Elaboration article discusses each checklist item and gives methodological background and published examples of transparent reporting. The STROBE checklist is best used in conjunction with this article (freely available on the Web sites of PLoS Medicine at http://www.plosmedicine.org/, Annals of Internal Medicine at http://www.annals.org/, and Epidemiology at http://www.epidem.com/). Information on the STROBE Initiative is available at http://www.strobe-statement.org.
