# Supplementary material for: KSHV K15 genetic heterogeneity is associated with Kaposi sarcoma aggressiveness through modulation of the host inflammatory microenvironment
Source: PLoS Pathog. 2026 Oct 7;22(10):e1014615. doi: 10.1371/journal.ppat.1014615 (PMC13660985; doi:10.1371/journal.ppat.1014615)
Supplement: Supplementary file 3 [file ppat.1014615.s001.docx]

**S1 Table.** Univariate Associations between 39 Clinical/Laboratory Parameters and K15 Genotypes

| Variables | | Total | K15 | | F/χ2 | P |
| --- | --- | --- | --- | --- | --- | --- |
|  |  |  | K15P | K15M |  |  |
| Comorbidities | Hypertension, n (%) | 52(33.3) | 8(20.0) | 39(36.4) | 3.622 | 0.057 |
|  | Diabetes Mellitus, n (%) | 28(17.9) | 8(20.0) | 19(17.8) | 0.098 | 0.755 |
|  | Coronary Artery Disease, n (%) | 35(22.4) | 6(15.0) | 26(24.3) | 1.478 | 0.224 |
|  | HIV Infection, n (%) | 23(14.7) | 11(27.5) | 11(10.3) | 6.784 | 0.009 |
|  | Lower Limb Edema, n (%) | 52(33.3) | 8(20.0) | 40(37.4） | 4.320 | 0.045 |
| Involvement | Only cutaneous, n (%) | 138(88.5) | 33(82.5) | 97(90.6) | - | 0.244 |
|  | Cutaneous and Mucosal, n (%) | 14(9.0) | 7(17.5) | 6(5.6) | - | 0.044 |
|  | Cutaneous and Visceral, n (%) | 6(3.8) | 0 | 5(4.7) | - | 0.324 |
|  | Cutaneous and extracutaneous, n (%) | 2(1.3) | 0 | 1(0.9) | - | 1.000 |
| Skin lesions on body surface area | ≤1%, n (%) | 42(26.9) | 7(17.5) | 33(30.8) | 2.616 | 0.106 |
|  | 1%-5%, n (%) | 83(53.2) | 24(60.0) | 57(53.3) | 0.533 | 0.465 |
|  | ≥5%, n (%) | 31(19.9) | 9(22.5) | 17(15.9) | 0.874 | 0.350 |
| Stage | localized stage, n (%) | 76(48.7) | 15(37.5) | 56(52.3) | 2.567 | 0.109 |
|  | Disseminated stage, n (%) | 80(51.3) | 25(62.5) | 51(47.7) |  |  |
| Cutaneous Lesion | single, n (%) | 14(9.0) | 2(5.0) | 11(10.3) | - | 0.515 |
|  | multiple, n (%) | 142(91.9) | 38(95.0) | 96(89.7) |  |  |
| Complete Blood Count | White blood cell count, median [IQR] | 6.25[5.00-7.84] | 6.17[4.45-7.90] | 6.44[5.02-7.84] | -0.455 | 0.649 |
|  | Haemoglobin, median [IQR] | 136.0[122.0-148.0] | 131[113.5-146.5] | 137.0[122.0-150.0] | -1.539 | 0.124 |
|  | Platelet count, median [IQR] | 223.0[178.0-259.0] | 217.0[167.5-244.0] | 225.0[185.0-265.0] | -1.676 | 0.094 |
|  | Neutrophil count, median [IQR] | 3.89[3.08-4.86] | 3.47[2.73-4.08] | 4.14[3.20-5.13] | -2.988 | 0.003 |
|  | Lymphocyte count, median [IQR] | 1.58[1.28-1.98] | 1.89[1.30-2.30] | 1.54[1.27-1.85] | -2.259 | 0.024 |
|  | Monocyte count, median [IQR] | 0.53(0.39-0.68) | 0.56[0.35-0.82] | 0.52[0.39-0.68] | -0.146 | 0.884 |
|  | Eosinophil count, median [IQR] | 0.17(0.09-0.35) | 0.17[0.09-0.44] | 0.17[0.08-0.33] | -0.560 | 0.576 |
|  | Basophil count, median [IQR] | 0.03[0.02-0.05] | 0.03[0.01-0.05] | 0.03[0.02-0.04] | -0.102 | 0.919 |
|  | Albumin, median [IQR] | 36.7[34.2-39.8] | 36.9[34.0-39.85] | 36.6[34.0-39.1] | -0.011 | 0.991 |
| Inflammatory Markers | SII, median [IQR] | 566.8[358.6-734.0] | 392.8[223.7-572.6] | 622.6[433.5-859.3] | -4.033 | <0.001 |
|  | PLR, median [IQR] | 140.2[106.4-183.0] | 111.5[87.3-158.3] | 146.5[114.04-199.3] | -3.325 | <0.001 |
|  | NLR, median [IQR] | 2.52[1.84-3.26] | 1.94[1.46-2.87] | 2.61[2.13-3.34] | -3.271 | 0.001 |
|  | LMR, median [IQR] | 3.08[2.05-4.14] | 3.25[2.36-4.22] | 3.03[1.82-4.08] | -1.175 | 0.240 |
|  | NPR, median [IQR] | 0.02[0.01-0.02] | 0.02[0.02-0.02] | 0.02[0.01-0.02] | -0.510 | 0.610 |
|  | ESR, median [IQR] | 18.6[12.0-23.4] | 18.6[12.3-22.0] | 18.6[11.0-24.0] | -0.118 | 0.906 |
|  | Interleukin-6, median [IQR] | 13.5[12.0-15.3] | 12.9[11.7-14.4] | 13.6[12.0-15.6] | -1.728 | 0.084 |
|  | Procalcitonin, median [IQR] | 1.35[0.88-1.85] | 1.40[0.94-1.85] | 1.34[0.74-1.88] | -0.714 | 0.475 |
|  | CRP, median [IQR] | 7.17[3.90-15.06] | 6.24[3.00-13.11] | 18.29[4.39-16.21] | -1.669 | 0.095 |
| Lymphocyte Subsets | CD4 T-Cell Count, median [IQR] | 607.5[498.0-653.0] | 604.5[392.0-624.6] | 607.7[543.9-655.0] | -0.862 | 0.389 |
|  | CD4/CD8, median [IQR] | 1.05[1.01-1.38] | 1.04[0.86-1.23] | 1.05[1.02-1.44] | -1.374 | 0.169 |

Abbreviations: Cutaneous and extracutaneous refer to simultaneous involvement of the skin and visceral and mucosal tissues; SII, Systemic Immune-Inflammation Index; PLR, Platelet-to-Lymphocyte Ratio; NLR, Neutrophil-to-Lymphocyte Ratio; ESR: Erythrocyte Sedimentation Rate; Statistical significance is indicated by boldface type.
