# Supplementary material for: KSHV K15 genetic heterogeneity is associated with Kaposi sarcoma aggressiveness through modulation of the host inflammatory microenvironment
Source: PLoS Pathog. 2026 Oct 7;22(10):e1014615. doi: 10.1371/journal.ppat.1014615 (PMC13660985; doi:10.1371/journal.ppat.1014615)
Supplement: Supplementary file 4 [file ppat.1014615.s002.docx]

**S2 Table.** Distribution of Primary Treatment Modalities Across KSHV K15 Genotypes.

| Treatment Modality | Entire Cohort (N=147) | K15P (n=40) | K15M (n=107) | *P-value* |
| --- | --- | --- | --- | --- |
| Systemic or Combined Therapy | 119(81.0%) | 28(70.0%) | 91(85.0%) | 0.104 |
| Local Therapy | 13(8.8%) | 6(15.0%) | 7(6.5%) |  |
| Observation / Supportive | 15(10.2%) | 6(15.0%) | 9(8.4%) |  |

Note:  Data are presented as n (%). Systemic or combined therapy includes immunotherapy alone or in combination with chemotherapy, radiotherapy, or surgery. Local therapy only includes radiotherapy, surgical excision, or cryotherapy without systemic agents. No active treatment refers to supportive care or observation. P-value was calculated using the Fisher-Freeman-Halton exact test due to small cell counts.
