# Supplementary material for: KSHV K15 genetic heterogeneity is associated with Kaposi sarcoma aggressiveness through modulation of the host inflammatory microenvironment
Source: PLoS Pathog. 2026 Oct 7;22(10):e1014615. doi: 10.1371/journal.ppat.1014615 (PMC13660985; doi:10.1371/journal.ppat.1014615)
Supplement: Supplementary file 5 [file ppat.1014615.s003.docx]

**S3 Table.** Causal Mediation Analysis, Directionality, and Dose-Response Assessment Linking K15M Genotype to Lower Limb Edema.

| Path & Analysis Strategy | Total Effect β (95% CI); P | Direct Effect (ADE)β (95% CI); P | Indirect Effect (ACME)β (95% CI); P | Proportion Mediated |
| --- | --- | --- | --- | --- |
| Panel A: Comparative Mediation Analysis of Inflammatory Markers | | | | |
| Mediator: SII (Primary) | 0.186 (0.014, 0.337); 0.038 | 0.130 (-0.044, 0.285); 0.130 | 0.057 (0.011, 0.117);0.009⋆⋆ | 29.0% |
| Mediator: NLR | 0.179 (-0.005, 0.331); 0.052 | 0.141 (-0.042, 0.296); 0.118 | 0.038 (-0.007, 0.009);0.106 | 18.7% |
| Mediator: PLR | 0.179 (0.000, 0.328); 0.049 | 0.137(-0.039, 0.290); 0.118 | 0.042(0.004 0.095);0.029⋆ | 22.1% |
| Panel B: Sensitivity Analysis for SII (Adjusting for CD4 Counts) | | | | |
| Model A: Core Model | 0.186 (0.014, 0.337); 0.038 | 0.130 (-0.044, 0.285); 0.130 | 0.057 (0.011, 0.117);0.009⋆⋆ | 29.0% |
| Model B: Adjusted for CD4 | 0.174 (0.006, 0.321); 0.045 | 0.116 (-0.056, 0.269); 0.172 | 0.058 (0.013, 0.113);0.010⋆ | 31.3% |
| Panel C: Directionality Validation (Reverse Mediation) | | | | |
| Forward (Primary):  K15 →SII → Edema | 0.186 (0.014, 0.337); 0.038 | 0.130 (-0.044, 0.285); 0.130 | 0.057 (0.011, 0.117);0.009⋆⋆ | 29.0% |
| Reverse (Validation):  K15 →Edema →SII | 300.6(118.1,491.4);0.001 | 248.5(89.6,408.6);0.002 | 52.1(-5.1,137.4);0.075 | 16.3% |
| Panel D. Quantitative assessment of dose-response relationships between SII and edema. | | | | |
| Analysis | OR (95% CI) per 500-unit SII increase | | P | Interpretation |
| Overall dose-response | 1.73 (1.22-2.63) | | 0.0057 | Significant |
| Stratified: K15P genotype | 2.02 (0.55-8.00) | | 0.2705 | Non-significant |
| Stratified: K15M genotype | 1.58 (1.11-2.48) | | 0.0246 | Significant |
| Genotype × SII interaction | - | | 0.7185 | Non-significant |

Panel A–C present the mediation framework. Statistical significance is indicated by asterisks: * P < 0.05, ** P < 0.01. Estimates were obtained using quasi-Bayesian Monte Carlo approximation (5,000 simulations). Panel C confirms the directionality (Inflammation → Edema). Panel D provides quantitative validation, showing the risk of edema per 500-unit increase in SII stratified by genotype to corroborate the mediation pathway.

Abbreviations: ACME, average causal mediation effects (indirect effect); ADE, average direct effects; CI, confidence interval; ns, not significant; OR, odds ratio; SII, systemic immune-inflammation index.
