# Supplementary material for: KSHV K15 genetic heterogeneity is associated with Kaposi sarcoma aggressiveness through modulation of the host inflammatory microenvironment
Source: PLoS Pathog. 2026 Oct 7;22(10):e1014615. doi: 10.1371/journal.ppat.1014615 (PMC13660985; doi:10.1371/journal.ppat.1014615)
Supplement: Supplementary file 6 [file ppat.1014615.s004.docx]

**S4 Table.** Sensitivity Analysis of Progression-Free Survival Under Varying Censoring Scenarios.

| Scenario | Maximum Interval | Number of Patients/ Events | Median PFS, months (95% CI) K15P/K15M | Log-rank P | HR (95% CI) |
| --- | --- | --- | --- | --- | --- |
| Primary | 24 months | 147/67 | 40 (14.5,65.5)/26 (18.4,33.6) | 0.017 | 2.051(1.112,3.783) |
| Conservative | 12 months | 147/67 | 40 (5.9,74.1)/24 (15.4,32.6) | 0.021 | 2.003(1.085,3.696) |
| Liberal | 36 months | 147/67 | 46 (34.7,57.3)/34 (22.1,45.9) | 0.025 | 1.965(1.067,3.616) |
| Excluded | Patients excluded | 105/65 | 25 (4.0,46.0)/11 (7.3,14.7) | 0.042 | 1.889(0.998,3.576) |

The table supplements Figure 3C by providing the median PFS (months) and patient counts for each censoring scenario to verify result robustness.

Abbreviations: CI, confidence interval; HR, hazard ratio; PFS, progression-free survival.
