# Supplementary material for: KSHV K15 genetic heterogeneity is associated with Kaposi sarcoma aggressiveness through modulation of the host inflammatory microenvironment
Source: PLoS Pathog. 2026 Oct 7;22(10):e1014615. doi: 10.1371/journal.ppat.1014615 (PMC13660985; doi:10.1371/journal.ppat.1014615)
Supplement: Supplementary file 7 [file ppat.1014615.s005.docx]

**S5 Table.** Associations of KSHV K15 Genotypes with Clinical, Inflammatory, and Prognostic Features in the Classic KS Subgroup.

| Features and Endpoints | Statistical Model | K15P (n = 25) | K15M (n = 90) | Effect Size (95% CI) | P value |
| --- | --- | --- | --- | --- | --- |
| Inflammatory Indices |  |  |  |  |  |
| SII | Wilcoxon rank-sum test | 495.0 [277.0–575.0] | 616.0 [441.0–816.0] | — | 0.003 |
| NLR | Wilcoxon rank-sum test | 1.94 [1.69–2.63] | 2.56 [2.14–3.23] | — | 0.006 |
| Clinical Phenotypes |  |  |  |  |  |
| Lower limb edema | Univariable logistic regression | Ref | — | OR = 1.91 (0.38–7.86) | 0.386 |
| Lower limb edema | Multivariable logistic regression † | Ref | — | aOR = 2.27 (0.41–10.56) | 0.304 |
| Prognostic Target |  |  |  |  |  |
| PFS | Multivariable Cox regression † | Ref | — | aHR = 1.44 (0.73–2.85) | 0.297 |

†Adjusted for age, sex, and tumour stage. Values are median [IQR] unless otherwise stated.

Note:Analyses were strictly confined to the Classic KS subgroup with available K15 genotyping data (N = 115). Continuous inflammatory parameters (SII, NLR) are presented as median [interquartile range, IQR]. The K15P genotype served as the reference (Ref) category for all regression models.
† Multivariable models (logistic regression for lower limb edema and Cox proportional hazards regression for PFS) were adjusted for clinically significant covariates including age, sex, and tumor stage.
Abbreviations: CI, confidence interval; OR, odds ratio; aOR, adjusted odds ratio; aHR, adjusted hazard ratio; Ref, reference;SII, systemic immune-inflammation index;NLR,neutrophil-to-lymphocyte Ratio;PFS,Progression-free survival .
