# Supplementary material for: KSHV K15 genetic heterogeneity is associated with Kaposi sarcoma aggressiveness through modulation of the host inflammatory microenvironment
Source: PLoS Pathog. 2026 Oct 7;22(10):e1014615. doi: 10.1371/journal.ppat.1014615 (PMC13660985; doi:10.1371/journal.ppat.1014615)
Supplement: Supplementary file 8 [file ppat.1014615.s006.docx]

**S6 Table.** Variable selection process and multicollinearity assessment for the prognostic model.

| Variable | Step 1: Optimization | Step 2: Association Screen | | Step 3: LASSO Selection | Step 4: Model Stability |
| --- | --- | --- | --- | --- | --- |
|  | MaxStat Cutoff | Univariate HR (95% CI) | P | LASSO Coeff (λmin) | VIF Score |
| K15 Genotype | - | 2.09 (1.13-3.85) | 0.019 | 0.307 | 1.05 |
| NLR | 2.48 | 0.59 (0.36-0.98) | 0.040 | -0.332 | 1.02 |
| CD4 Count | 792.69 | 4.74 (2.50-9.00) | <0.001 | 1.136 | 1.03 |
| LMR | 1.27 | 2.73 (1.16-6.42) | 0.021 | 0.555 | 1.01 |
| PCT | 0.74 | 2.54 (1.49-4.33) | <0.001 | 0.620 | 1.09 |
| ESR | 19.00 | 1.62 (0.99-2.66) | 0.057 | 0.114 | - |
| PLR | 118.86 | 0.57 (0.33-0.98) | 0.041 | - | - |
| SII | 571.82 | 0.63 (0.38-1.04) | 0.069 | - | - |
| Age | 69.00 | 1.21 (0.68-2.15) ^a^ | 0.526 | - | - |
| Visceral Inv. | - | 2.57 (0.93-7.13) | 0.070 | 0.547 | 1.07 |

Step 1: Optimal cutoffs were determined using MaxStat. Step 3: LASSO coefficients represent the variable importance at the optimal lambda ($\lambda_{\min}$). Step 4: VIF scores < 2.0 indicate the absence of multicollinearity.Symbols: "-" indicates not applicable (e.g., variable excluded by LASSO or categorical variable not requiring cutoff).a For age, the optimal MaxStat cutoff was 69 years; however, the standard clinical stratification (<55 vs. ≥55 years) was used for the final modeling to maintain clinical applicability.

Abbreviations: CI, confidence interval; HR, hazard ratio; LASSO, least absolute shrinkage and selection operator; LMR, lymphocyte-to-monocyte ratio; MaxStat, maximally selected rank statistics; NLR, neutrophil-to-lymphocyte ratio; PCT, procalcitonin

; PFS, progression-free survival; SII, systemic immune-inflammation index; VIF, variance inflation factor.
