# Supplementary material for: KSHV K15 genetic heterogeneity is associated with Kaposi sarcoma aggressiveness through modulation of the host inflammatory microenvironment
Source: PLoS Pathog. 2026 Oct 7;22(10):e1014615. doi: 10.1371/journal.ppat.1014615 (PMC13660985; doi:10.1371/journal.ppat.1014615)
Supplement: Supplementary file 9 [file ppat.1014615.s007.docx]

**S7 Table.** Primer sequences used for KSHV K15 genotyping via nested PCR.

| Target Genotype | PCR Round | Primer Name | Sequence (5′–3′) | Amplicon Size | Reference |
| --- | --- | --- | --- | --- | --- |
| K15 P-type | Round 1 | K15P-OF | TGCAGGCTTGGTCATGGGTTAC | 365bp | [16] |
|  |  | K15P-OR | GGGACCACGCYGCAATTAAATG |  |  |
|  | Round 2 (Nested) | K15-3C | ACGCATACATGTACTGCCAC | 285 bp | [14] |
|  |  | K15-4C | CTTTGATATTGCCAGTGG TG |  |  |
| K15 M-type | Round 1 | K15M-OF | TGTTGGTTGCAATGCTTAGGTG | 486bp | [8] |
|  |  | K15M-OR | GCCTTTGCCAGTTGGAGTTTC |  |  |
|  | Round 2 (Nested) | LGH 2473 | CATGCAGCGAGCTTGAGA | 370 bp | [8] |
|  |  | LGH 2474 | CTTTGAGTACTGTTTGTG |  |  |
